# Editorial: Thymus function and aging: A focus on thymic epithelial cells

**DOI:** 10.3389/fimmu.2022.1003490

**Published:** 2022-08-15

**Authors:** Valentin P. Shichkin, Maria Pia Felli, Isabella Screpanti, Mariastefania Antica

**Affiliations:** ^1^ OmniFarma, Kyiv, Ukraine; ^2^ Department of Experimental Medicine, Sapienza University of Rome, Rome, Italy; ^3^ Department of Molecular Medicine, Sapienza University of Rome, Rome, Italy; ^4^ Division of Molecular Biology, Rudjer Boskovic Institute, Zagreb, Croatia

**Keywords:** thymus regeneration and development, thymic stem cells, thymic epithelial cells, T cell development, thymus regeneration

The thymus is an unique organ in its ability to support the maturation of phenotypically and functionally distinct T cell sublineages and innate immune cells, which carry out multiple tasks to keep the organism healthy. Entering the thymus, lymphocyte precursors interact with the thymic stromal compartment mainly built of different thymic epithelial cells (TECs) and other non-lymphoid cells comprising the thymic microenvironment for T cell development (1). From the enormous variety of produced T lymphocytes, only a minor part can survive a rigorous checkpoint control and selection during this crosstalk (2). However, some physiological factors, including aging, stress, and pregnancy, as well as medical procedures such as thymectomy and chemo/radiotherapy, can harm the thymus function, which is associated with the decline of immune function and the risk of tumors and infectious and autoimmune diseases.

In this Research Topic are collected the efforts of many research groups in trying to overcome the thymus aging/injury problem by applying different regenerative or thymus replacement strategies. The basis of these is the epithelial compartment, in particular, the thymic epithelial stem cells (TESCs) as the target cells to stimulate thymus recovery *in vivo* or for growing thymus-replacing organoids *in vitro* (3, 4). Several research groups have described TESCs in the embryonal (5) and adult (6–8) mouse thymus, which were identified as the bipotent TEC progenitors differentiating into cortical (c) and medullary (m) TEC lineages (5–8). Progress in the identification of different TEC subtypes as well as other subtypes of thymic cells in the last several years was significant, especially with applying the single-cell RNA-sequencing technology (9–11).

T cell development in the thymus depends on Notch signalling (12) induced by the interaction of Notch1, present on immigrant cells, with a Notch ligand, delta-like ligand (Dll) 4, on the thymic epithelial cells. Hirano et al. propose a hypothesis that in the thymic environment of ancestral vertebrates, where the thymus first appeared, primarily functions Dll1 and Notch2. The authors confirmed that Dll1 cooperates with Notch2 in T cell development in the murine thymus. Their results support the hypothesis that Dll1 regulates T cell development *via* Notch1 and/or Notch2 in the thymus of cartilaginous fishes. In the authors’ opinion, during the evolutionary process, Dll4 replaces Dll1 in the induction of thymic Notch signalling, constituting an environment in the thymus suitable for immigrant cells bearing Notch1.

Initial studies, recently confirmed by genetic approaches, have extended the role of Notch signalling to the epithelial compartment of the thymus, showing that active Notch contributes to TEC development during embryonic life. García-León et al. showed that *in vivo* Notch activation is not confined to embryonic TECs, but Notch signalling, likely mediated through the Notch1 receptor, is induced as well in postnatal TECs mainly located in the medulla (mTECs). In both human and mouse thymus, numbers of mTECs showing Notch activation increased significantly with age, suggesting a conserved role for Notch in postnatal TEC homeostasis during aging. TEC-specific abrogation of Notch signalling disrupted the medullary thymic microenvironment and accelerated thymus atrophy. These data uncover a new role for Notch1 signalling in the control of adult mTEC homeostasis.

Differentiating the human pluripotent stem cells towards thymic endoderm, Sun et al. identified a new population of FOXN1^+^EPCAM^+^CD90^+^ triple-positive TEC progenitors. They confirmed the existence of similar cells in cultures of neonatal human TECs. Also, they showed that a subset of primary neonatal human TECs co-express a marker of mesenchymal cells CD90 and a TEC marker EPCAM that reflect the presence of a mesenchymal program in human TECs. This program was more expressed in cTECs. Their results reveal that human TECs possess a hybrid gene expression program comprising epithelial and mesenchymal elements.

Proper T cell function is paramount to health and homeostasis. However, it is unclear whether the thymic ability to support incoming progenitors is affected by aging and the associated thymic involution. Mohtashami et al. compared the ability of progenitor T cells to home to the thymus of young and old mice and determined whether progenitor T cells can help support T cell regeneration in a clinically relevant model of hematopoietic stem cell transplants (HSCT). They demonstrated that the adoptive transfer of *in vitro*-generated pro-T cells in aged mice accelerated thymic reconstitution after chemotherapy and gamma irradiation compared to HSCT alone. Accelerated T cell recovery was also observed in both old and young mice receiving both pro-T cells and HSCT.

A critical part of the processes associated with central tolerance occurs in the thymic medulla. It depends on the presence of various types of dendritic cells (DCs), B cells, and highly specialized mTECs. Cooperation between these cells is required to remove autoreactive T cells efficiently. This crosstalk is relevant not only during thymus organogenesis and T cell development but also promotes the recovery of the thymus functionality after injuries. Březina et al. in their review paper, highlight the current knowledge concerning the pathways by which self-antigens are presented in the thymus and how they lead to the establishment of tolerance. They also examined and discussed the possible molecular mechanisms underpinning cooperative antigen transfer. Finally, they discussed the current results related to distinct preferences of DC subsets in acquiring thymic epithelial cell-derived antigens.

Shichkin and Antica discuss cellular architecture and molecular factors essential for correct thymic function relating to T cell positive and negative selection and generation of naïve T cells. The authors summarize the current understanding of the development and function of TECs and other stromal cell populations, the signalling and transcriptional pathways underlying the intrathymic cell interaction, and T cell development concerning developing new strategies for restoring thymic function after damage. The authors accentuate populations of intrathymic stem cells (SCs), including epithelial SCs, mesenchymal SCs, and lymphoid progenitor cells. The particular focus is on their radioresistance and, thus, possible contribution to thymus recovery after injury with irradiation or chemotherapy.

Rosichini et al. analyzed signals involved in the crosstalk between TECs and hematopoietic cells. The authors’ primary focus is on how T cell signals regulate TEC function. The authors also discuss the relevance of these pathways in restoring thymic function and T cell immunity in experimental models and in the clinical setting.

Lagou et al. propose a fresh insight that chemotherapy-induced thymic involution, which is characterized by the extensive obliteration of the sensitive TEC compartment, can cause long-term defects in thymopoiesis and the establishment of diverse T cell pools of cancer survivors patients. Such delayed recovery of the T cell adaptive immunity may result in the prolonged disturbance of the cancer immunoediting mechanisms and lead to the development of persistent and mortal infections, inflammatory disorders, autoimmune precursor lesions, and second primary malignancies.

Finally, Iaiza’s et al. are focused on the involvement of long non-coding RNAs (lncRNAs) in the acquisition of malignant traits by neoplastic TECs, and describes the possible use of these molecules as targets for the design of novel therapeutic approaches specific for TECs. Furthermore, they discuss the involvement of lncRNAs in myasthenia gravis-related thymoma.

In summary, we anticipate that the articles in this *Frontiers Research Topic *will provide researchers with a valuable resource for understanding how we can improve the current and develop new strategies for thymus recovery/replacement and translate them to clinics.

## Author contributions

VS, MPF, IS, and MA wrote the editorial and invited authors to participate in the collection. All authors contributed to the article and approved the submitted version.

## Funding

MA was supported by grants from Croatian Science Foundation (IP-2020-02-2431); the Terry Fox Foundation Zagreb Run and Croatian League against Cancer; and the Scientific Centre of Excellence for Reproductive and Regenerative Medicine (Grant Agreement KK01.1.1.01.0008 that is funded by the European Union through the European Regional Development Fund). MPF was supported by Sapienza University 2021 (RP12117A63FBA27C). IS was supported by the Italian Ministry of University and Research - Dipartimenti di Eccellenza - L. 232/2016. IS and MPF were supported by grant THYMINNOVA (IP-2020-02-2431).

## Acknowledgments

VS wishes to thank Dr. Oleg Kurchenko, OmniFarma’s CEO and founder.

## Conflict of interest

Author Valentin Shichkin was employed by the company OmniFarma.

The remaining authors declare that the research was conducted in the absence of any commercial or financial relationships that could be construed as a potential conflict of interest.

## Publisher’s note

All claims expressed in this article are solely those of the authors and do not necessarily represent those of their affiliated organizations, or those of the publisher, the editors and the reviewers. Any product that may be evaluated in this article, or claim that may be made by its manufacturer, is not guaranteed or endorsed by the publisher.
